# A genome-wide study to identify genes responsible for oviduct development in chickens

**DOI:** 10.1371/journal.pone.0189955

**Published:** 2017-12-27

**Authors:** Manman Shen, Liang Qu, Meng Ma, Taocun Dou, Jian Lu, Jun Guo, Yuping Hu, Xingguo Wang, Yongfeng Li, Kehua Wang, Ning Yang

**Affiliations:** 1 Jiangsu Institute of Poultry Science, Chinese Academy of Agricultural Science, Yangzhou, China; 2 College of Animal Science and Technology, Yangzhou University, Yangzhou, China; 3 College of Animal Science and Technology, China Agricultural University, Beijing, China; Wageningen UR Livestock Research, NETHERLANDS

## Abstract

Molecular genetic tools provide a method for improving the breeding selection of chickens (*Gallus gallus*). Although some studies have identified genes affecting egg quality, little is known about the genes responsible for oviduct development. To address this issue, here we used a genome-wide association (GWA) study to detect genes or genomic regions that are related to oviduct development in a chicken F_2_ resource population by employing high-density 600 K single-nucleotide polymorphism (SNP) arrays. For oviduct length and weight, which exhibited moderate heritability estimates of 0.35 and 0.39, respectively, chromosome 1 (GGA1) explained 9.45% of the genetic variance, while GGA4 to GGA8 and GGA11 explained over 1% of the variance. Independent univariate genome-wide screens for oviduct length and weight detected 69 significant SNPs on GGA1 and 49 suggestive SNPs on GGA1, GGA4, and GGA8. One hundred and fourteen suggestive SNPs were associated with oviduct length, while 73 SNPs were associated with oviduct weight. The significant genomic regions affecting oviduct weight ranged from 167.79–174.29 Mb on GGA1, 73.16–75.70 Mb on GGA4, and 4.88–4.92 Mb on GGA8. The genes *CKAP2*, *CCK*AR, *NCAPG*, *IGFBP3*, and *GORAB* were shown to have potential roles in oviduct development. These genes are involved in cell survival, appetite, and growth control. Our results represent the first GWA analysis of genes controlling oviduct weight and length. The identification of genomic loci and potential candidate genes affecting oviduct development greatly increase our understanding of the genetic basis underlying oviduct development, which could have an impact on the selection of egg quality.

## Introduction

Egg production and egg quality, as economic traits, play important roles in the poultry industry. Increasing the level of egg production to 500 by 100 weeks of age is an ultimate goal; however, as hens grows old, the albumen becomes thin and eggshell quality decreases sharply. Additionally, the frequency of abnormal eggs increases, which reduces the number of high-quality eggs. The oviduct is defined as having five segments: the infundibulum, magnum, isthmus, uterus, and vagina. After oviposition, the F1 follicle is captured by the infundibulum, and the albumen is formed in the oviduct magnum, while the eggshell is produced in the uterus [1]. Finally, egg output is regulated by the vagina. The oviduct is a complex biological pipeline in which hormones orchestrate numerous biochemical and cellular changes [2], mainly involving protein secretion and eggshell formation. Studies by Williams [3, 4] showed that albumen production is correlated positively to oviduct mass and a larger oviduct may produce high-quality eggs. Thus, it is an important organ that plays a key role in egg quality.

The eggs laid by oviparous chickens (*Gallus gallus*), compared with viviparous animals, must contain all the nutrients required to sustain embryonic development. Thus, the development of the oviduct will directly affect embryonic and chick development [5]. Because it is more time-consuming and expensive to produce human therapeutic proteins in industrial bioreactors, the demand for the development of transgenic animals as bioreactors is increasing [6]. The oviduct of transgenic hens may have advantages over mammalian expression systems as a bioreactor to provide potentially new therapeutic proteins [7], as laying hens over 2 years of age suffer from fibroid tumors that are similar to human fibroid tumors, thereby making them a good animal disease model [8, 9].

Despite many reports of hyperplasia and hypertrophy [10] maintaining the sustained fertility [11] of the oviduct of hens during their reproductive period, little information is available regarding the genetic architecture of the oviduct mass and length at the late laying period. With the development of molecular biology techniques, research into quantitative traits has made extensive progress. Such techniques are often used in genome-wide association studies (GWASs) to detect genes that are associated with quantitative traits [12, 13]. The purpose of the present study was to identify putative genomic regions and candidate genes associated with oviduct mass and length using an F2 resource population.

## Materials and methods

Our experiments complied with the regulations and guidelines for experimental animals established in the Guidelines for Experimental Animals, Ministry of Science and Technology (Beijing, China). Animal experiments were permitted by the Institutional Animal Care and Use Committees at the Poultry Institute, Chinese Academy of Agricultural Science, Yangzhou, China (permit number: JPIAE 2011–0005), and the Animal Ethics Committee of China Agricultural University, Beijing, China (permit number: SYXK 2007–0023).

### Animals and data

The F2 resource population in our experiment was described in previous reports [13–17]. It originated from a cross between White Leghorn (WL) and Dongxiang Blue-shelled (DX) chickens. WL layers produce more eggs than DX layers. The two lines differ in various traits, including morphological, physiological, and production traits, such as albumen weight, albumen height, eggshell weight, and the yolk proportion [18]. The F2 resource population was constructed by 49 half-sib and 590 full-sib families, with a WL:DX ratio of 25 males to 407 females, and a DX:WL ratio of 24 males to 235 females, by the F1 population in a single hatch. The F1 population consisted of 552 individuals, and 1,029 chicks were generated from six DX males and six WL males that were initially mated with 80 WL females and 133 DX females, respectively. The birds were vaccinated for Marek’s disease after hatching and reared under artificial illumination during the first week. When the hens reached 16 weeks of age, they were transferred to single cages and the amount of light was increased 1h/week until a 16 h light/8 h dark photoperiod was attained, and they were provided food comprising 165 g crude protein/kg and 11.5 MJ metabolizable energy/kg and water *ad libitum* at the Jiangsu Institute of Poultry Science, Yangzhou, China. Birds belonging to the F2 resource population were decapitated at 72 weeks of age after they were rendered unconscious by 60%–70% carbon dioxide. Then, their oviducts were isolated, weighed, and their length was measured.

Phenotypic data were computed by the MEANS procedure of SAS software using all available records. Only values that varied by +3 or −3 standard deviations from the mean were removed from the subsequent analysis. The RANK procedure in SAS was used for rank-based inverse normal transformations to convert trait deviations to normality.

### Genotyping and quality control

Blood samples were drawn from the wing vein, and DNA was extracted by the standard phenol/chloroform method and genotyped with the 600 K SNP Array (Affymetrix, Inc., Santa Clara, CA, USA). Detailed information regarding genotyping and quality control procedures is available in previous studies [13, 14, 16, 17]. Samples were excluded if the call rate was ≤ 97%, the dish quality control was ≤ 0.82, the Hardy–Weinberg equilibrium allele frequency, *p*, was < 1×10^−6^, the minor allele frequency was < 5%, and the imputation quality score, *R*^2^, was ≤ 0.5, as determined using the software Affymetrix Power Tools v1.16.0 (APT) (http://affymetrix.com/), the PLINK v1.90 program [19], and the BEAGLE v4.0 package. After quality control measures, 435,867 single-nucleotide polymorphisms (SNPs) were used in the downstream GWA analysis.

### Statistical analyses

Initially, a principal component analysis was used to adjust for the influence of spurious associations resulting from a hidden population stratification or the presence of cryptic relatedness in the analyzed dataset using a GWA matrix based on marker information. Furthermore, a test of thresholds for genome-wide significant/suggestive associations was performed using the simpleM method [20]. After a Bonferroni adjustment, a total of 59,308 effective independent tests were obtained. Hence, the genome-wide significant and suggestive *P*-values were 8.43 × 10^−7^ (0.05/59,308) and 1.69 × 10^−5^ (1.00/59,308), respectively.

The effect of genotype on the oviduct was first estimated from a single-traits analysis, which was conducted with the valid samples and SNPs using the GEMMA v0.94 package [21]. To determine the significance level, the *P*-value was derived by a Wald test using the equation:

y=Wα+xβ+μ+ε

Where **y** is a vector of phenotypic values for *n* individuals; **W** is a matrix of covariates (fixed effects with a column of 1s and the top five principle components (PCs)), **α**is a vector of the correspondence between the coefficients, including the intercept; **x** is a vector of the genotypes of the SNP marker; **β** is the effect size of the marker; **μ** is a vector of random individual effects; and **ε** is a vector of random errors.

Manhattan and quantile–quantile (Q–Q) plots were constructed by the “gap” and “qqman” packages [22] in the R project, respectively. The genomic inflation factor λ, which was derived from the GenABEL package, was used to assess the influence of population stratification [23].

### Linkage disequilibrium analysis

A linkage disequilibrium (LD) analysis was performed in Haploview v4.2 [24]. In this analysis, the block detection method adopted the Solid Spine algorithm, and a block was defined if there was strong LD between significant SNPs in a putative region, as evidenced by D′ ≥ 0.8. Finally, the independent association signals were confirmed.

### Contributions to phenotypic variance (CPVs) and gene annotation

First, we estimated the heritability from the eligible SNPs (*h*^*2*^_*snp*_) and genetic correlations using the software GCTA v1.24 [25]. Then, a genetic relationship matrix was produced to estimate the candidate loci CPVs for OW. The variance contributed by each chromosome was calculated using the GCTA program using the top five PCs as covariates, and the CPVs after the candidate loci were fitted as covariates. The annotated genes, based on version 5.0 of the *G*. *gallus* genome supported by the National Center for Biotechnology Information and Ensembl, located in or near leading loci were identified as potential candidate genes [26].

## Results

### Phenotype statistics and genetic parameters

Means, standard errors, the SNP-based (*h*^*2*^_*snp*_) heritability, and the genetic correlation for the oviduct are shown in **Table 1**. Oviduct length (OL) displayed a lower coefficient of variation (13.91%) than OW (23.37%). The confidence intervals of the CVs for OL and OW were 13.40%–14.43% and 22.50%–24.49%, respectively, which means that the data are sufficiently reliable for the GWA analysis. The phenotypic and genetic correlations were high between OL and OW. The estimated heritability for OL and OW were 0.354 and 0.392, respectively, which suggests that there is a considerable genetic contribution to these oviduct traits.

**Table 1:** Phenotypic descriptive statistics and genetic analysis of the oviduct.

Trait	No. of samples	Mean ± SD	CV (%)	CI (%)	Correlation (p/g)	*h*^*2*^_*snp*_
OL (cm)	1,469	48.18±6.70	13.91	13.40–14.43	0.614	0.354(0.044)
OW (g)		41.46±9.69	23.37	22.50–24.49	0.799(0.051)	0.392(0.043)

Abbreviations: Mean = arithmetic mean; SD = standard deviation; CV = coefficient of variance; CI = confidence interval of the CV; Correlation (p/g) = the p-phenotypic correlation, g-genetic correlation; h^2^_snp_ = SNP-based (*h*^*2*^_*snp*_) heritability. OL = oviduct length; OW = oviduct weight.

### Identification of candidate loci by a GWAS

**Fig 1:**
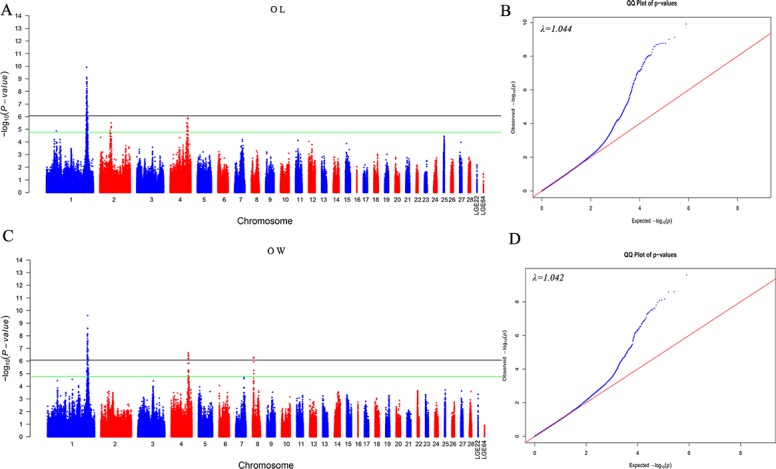
Manhattan (left) and Q–Q plots (right) of the observed *P* -values for oviduct traits. The Manhattan plot shows *P* values for the association of SNPs (*y*-axis) plotted against their chromosomal positions (*x*-axis). The black and green lines depict genome-wide significant (8.43 × 10^−7^) and suggestive significant (1.69 × 10^−5^) thresholds, respectively. For the Q–Q plot, the *x*-axis indicates the expected −log_10_-transformed *P* values, and the *y*-axis shows the observed −log_10_-transformed *P-*values. The genomic inflation factors (λ) are shown at the top left of the Q–Q plots.

**Fig 2:**
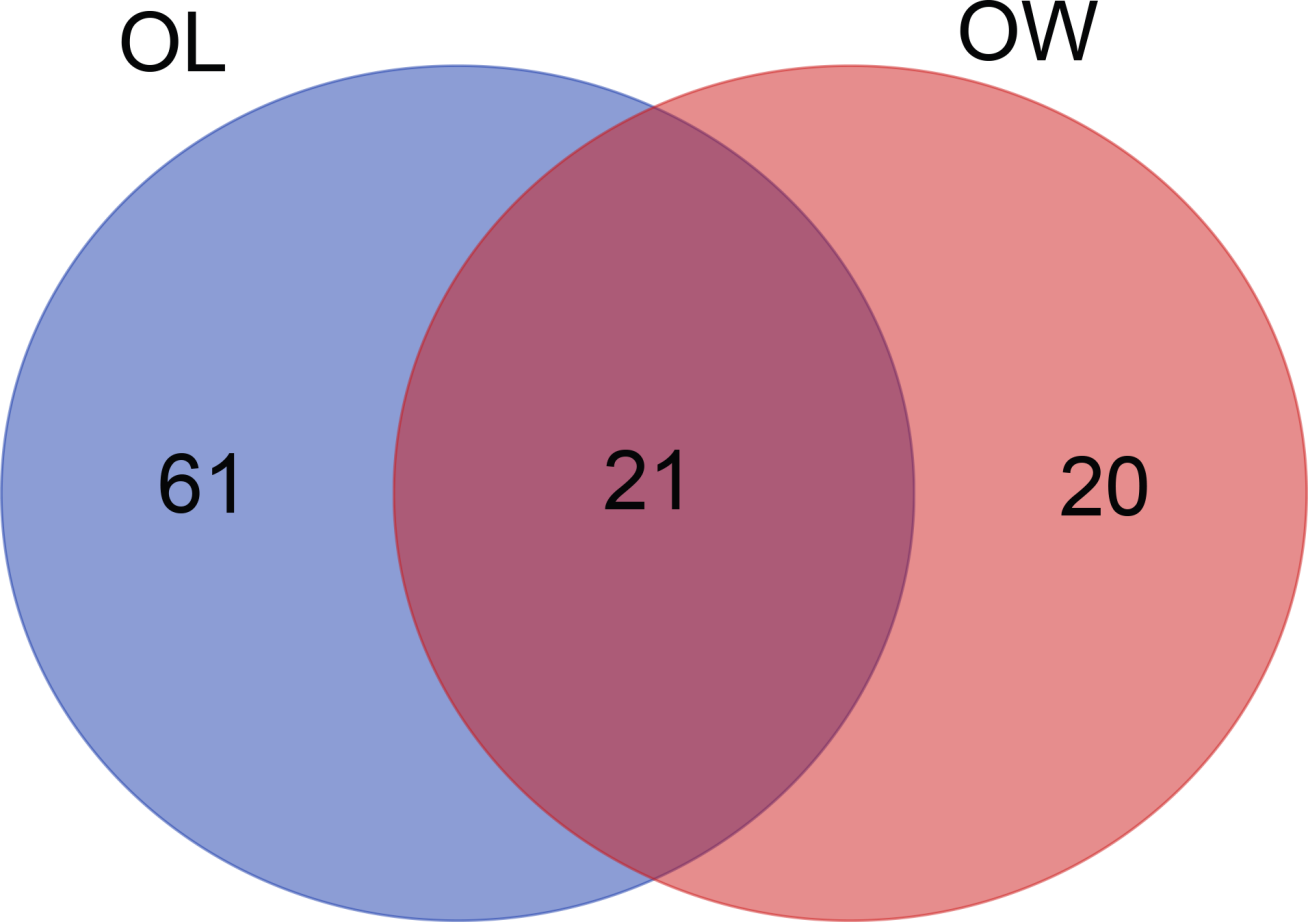
Venn diagram illustrating SNPs on GGA1 that are associated significantly with OW and OL.

The GWAS resulted in the detection of 82 and 52 SNPs that showed a significant association with OL and OW, respectively (**Table 2**). For OL, five and 17 SNPs located on *G*. *gallus* chromosome 2 (GGA2) and GGA4, respectively, reached the suggestive level. The significant SNPs located on GGA1 spanned from 169.19 to 175.05 Mb. For OW, eight and three SNPs located on GGA4 and GGA8, respectively, showed a significant association with OW. The available information for genome-wide significant SNPs is illustrated in **S1 and S2 Tables.** Manhattan and Q–Q plots are shown in **Fig 1**. Moreover, we constructed a Venn diagram for the significant loci on GGA1 because the genetic correlation was high (0.799) (**Fig 2**). We obtained 21 SNPs that were related to both OW and OL.

**Table 2:** Number and distribution of significant SNPs for oviduct traits.

Trait	OL			OW		
	GGA	No.	region	GGA	No.	region
Sig.	GGA1	82	169.19–175.05M	GGA1	41	168.38–171.87
				GGA4	8	74.03–76.70
				GGA8	3	4.92–4.95
Sug.	GGA1	79	168.38–175.02	GGA1	97	165.69–173.96
	GGA2	5	49.16–56.09	GGA4	20	72.76–75.70
	GGA4	17	72.75–77.37	GGA8	6	4.88–5.02

Abbreviations: Sig. = number of significant SNPs; Sug. = number of suggestive of SNPs; No. = number of SNPs

### Linkage analysis

**Fig 3:**
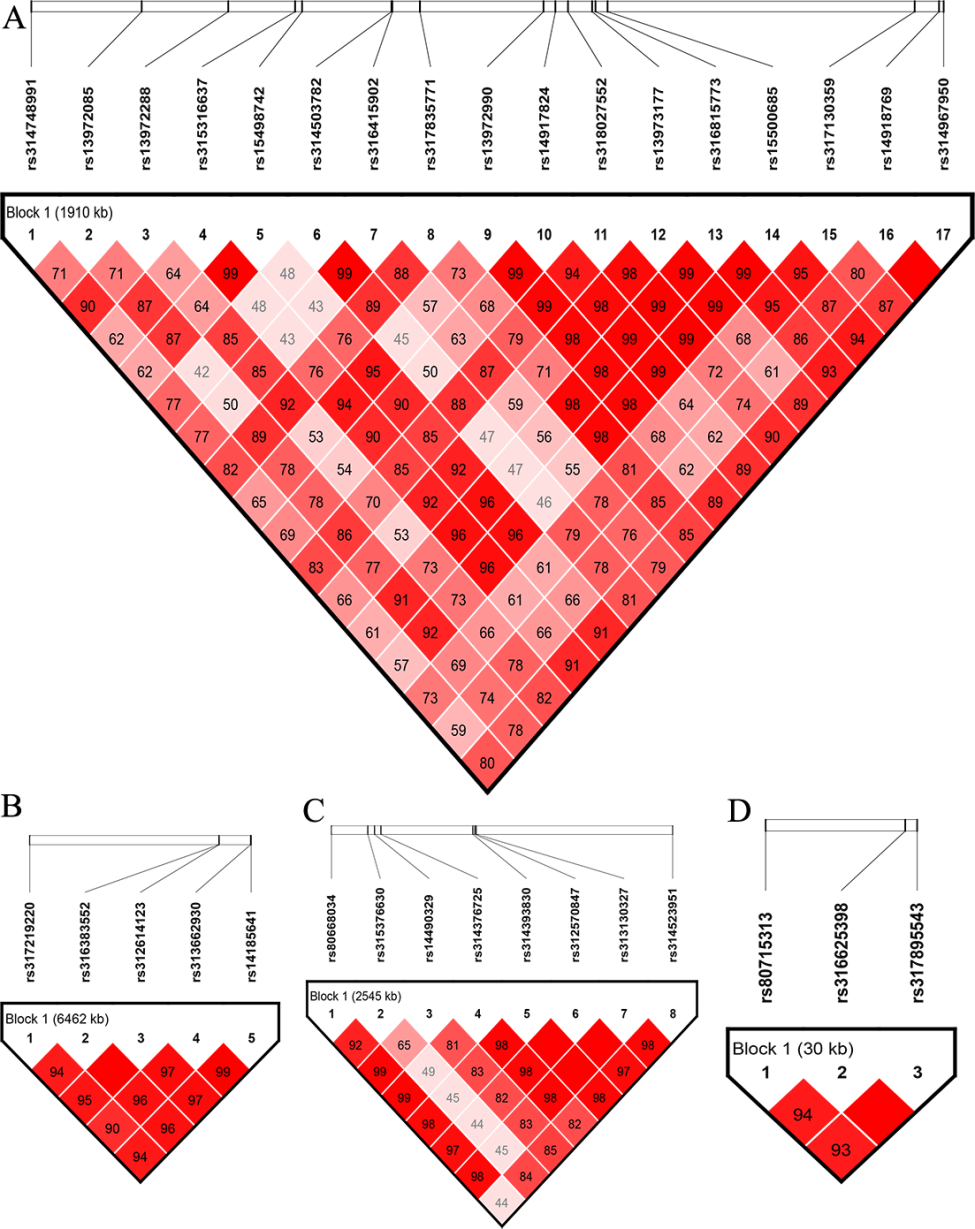
**Linkage analysis of the SNPs on GGA1, GGA4, GGA8, and GGA2** Plots A, B, C, and D show the linkage analysis for the SNPs on GGA1, GGA4, GGA8, and GGA2, respectively.

The significant loci on GGA1 were located in a region ranging from 168 to 171 Mb, corresponding to a region identified in our previous study [13], which showed that the SNPs in this region displayed an extremely strong LD status. The most significant locus, *rs318027552*, was chosen for further analysis. Then, we performed a linkage analysis for the significant loci on GGA4 and GGA8. The linkage analysis for the significant loci on GGA4 identified two haplotype blocks from 73.16 to 75.70 Mb **(Fig 3)**, and the three SNPs on GGA8 showed strong LD blocks. Consequently, *rs80668034* and r*s312570847* on GGA4, and *rs80715313* on GGA8 were chosen for further analysis. SNPs that were detected in GGA2 showed a suggestive association with OW, and they were subjected to an LD analysis because SNPs with a suggestive level may indicate important loci or they may overlap with SNPs identified in previous studies [27, 28]; after this step, *rs312614123* was chosen for further analysis.

### Estimated CPVs and identification of candidate genes

The significant loci were searched against the Ensembl database using the Basic Local Alignment Search Tool, and the results showed that they mapped to regions that were distant from the nearest known genes. The leading SNPs on GGA1, GGA4, GGA8, and GGA2 were mapped to the genes *CKAP2*, *CCKAR*, *NCAPG*, *GORAB*, and *IGFBP3* (**Table 3**). The SNPs on GGA1 accounted for more than 4% of the phenotypic variance of OW and OL, while the *rs80668034* locus that showed a suggestive association with OW explained 2.48% and 1.97% of the phenotypic variance of OL and OW. The locus *rs80715313* on GGA8 accounted for the lowest phenotypic variance for OL. Moreover, the suggestive locus on GGA2 accounted for 2.05% of the phenotypic variance of OW.

**Table 3:** Contributions of five mutations and genomic regions to oviduct trait.

SNP		rs318027552	rs80668034	rs312570847	rs80715313	rs312614123
GGA		1	4	4	8	2
**Position (bp)**		170318652	74034095	75146457	4917630	55157730
**Gene symbol**		CKAP2	CCKAR	NCAPG	GORAB	IGFBP3
**Location**		U88.12Kb	D331.64Kb	U1.31Mb	D7.34Kb	D38.59Kb
**EA/AA**		G/A	A/G	C/T	C/T	T/C
**MAF**		0.341	0.16	0.065	0.323	0.16
**OL**	beta (SE)	−0.315(0.049)	0.276(0.060)			−0.288(0.062)
	CPV (%)	4.45	1.97			2.05
	*P*-value	1.23E-10	5.76E-06			3.35E-06
**OW**	beta (SE)	−0.311(0.049)	0.313(0.060)	0.449(0.087)	0.210(0.042)	
	CPV (%)	4.23	2.48	2.24	1.71	
	*P*-value	2.52E-10	2.24E-07	2.91E-07	5.03E-07	

Abbreviations: SNP = single nucleotide polymorphism; GGA = *Gallus gallus* chromosome; EA = effect allele (minor allele); AA = alternative allele (major allele); MAF = minor allele frequency; OW = oviduct weight; OL = oviduct length; EA = estimated allelic substitution effect per copy of the effect allele; SE = standard error of the beta, which indicates the effect size of minor alleles; CPV = contribution to phenotypic variance (%).

**Fig 4:**
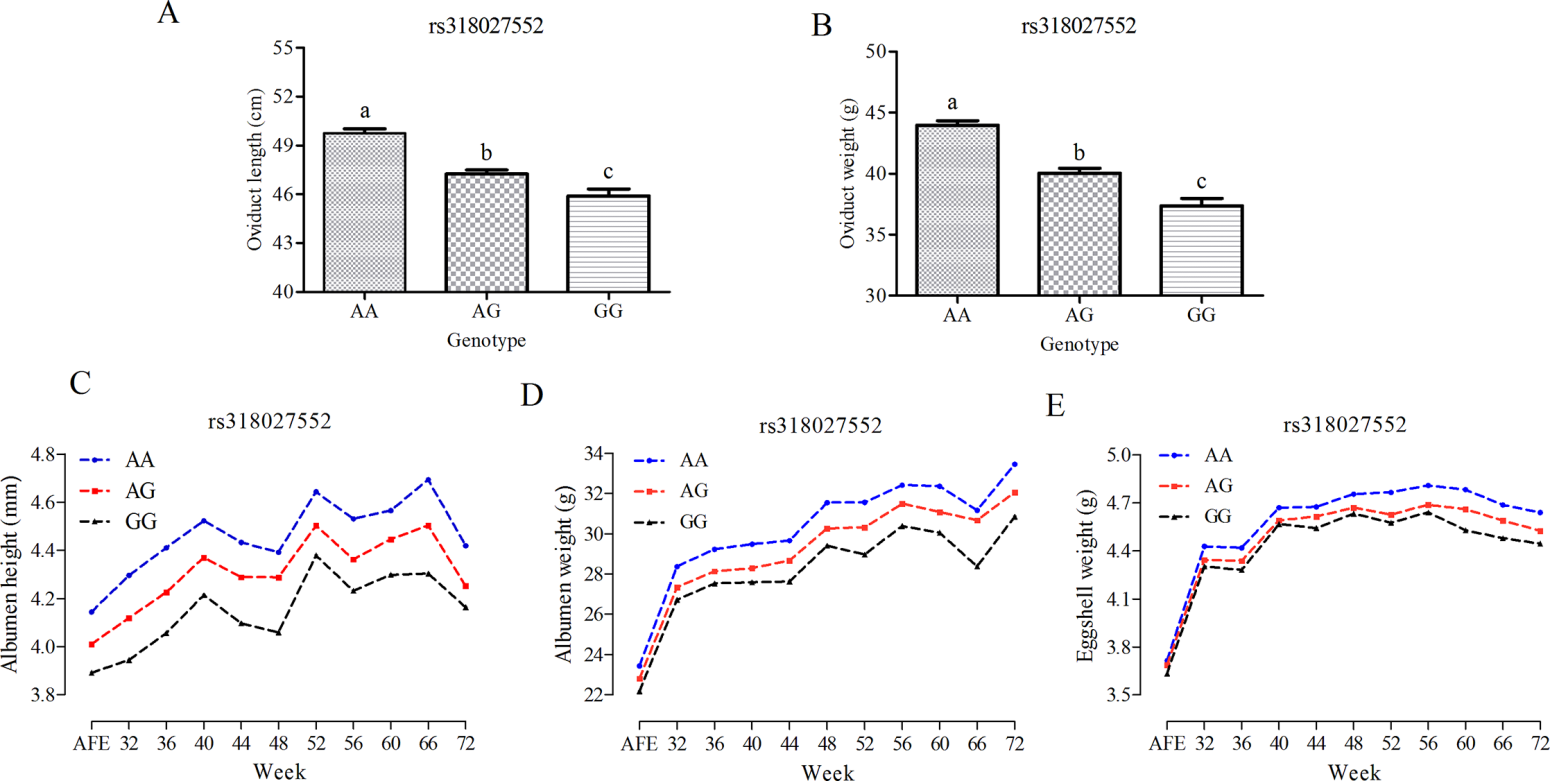
Genotype effect plot of *rs318027552* for oviduct traits and egg quality. Plots A and B describe the OL and OW phenotypes, respectively, of the three genotypes at *rs318027552*. Plots C, D, and E describe the phenotypes of egg quality phenotypes, including albumen height, albumen weight, and eggshell weight, respectively, of the three genotypes at *rs318027552*.

The three leading significant SNPs (*rs318027552*, *rs80668034*, and *rs80715313*) were analyzed to compare the actual phenotypes of the oviduct as well as egg quality differences among the three genotypes (Fig 4, S1 and S2 Figs). The results showed that the phenotype of the oviduct and the egg quality differed among the three genotypes of *rs315027552*, suggesting that this marker on GGA1 is also responsible for egg quality. Moreover, the GG genotype of *rs80668034* had a higher OW, albumen weight, and eggshell weight (**S1 Fig**), while the CC genotype of *rs80715313* had a lower OW and albumen height (**S2 Fig**).

### Partitioning of genetic variation

**Fig 5:**
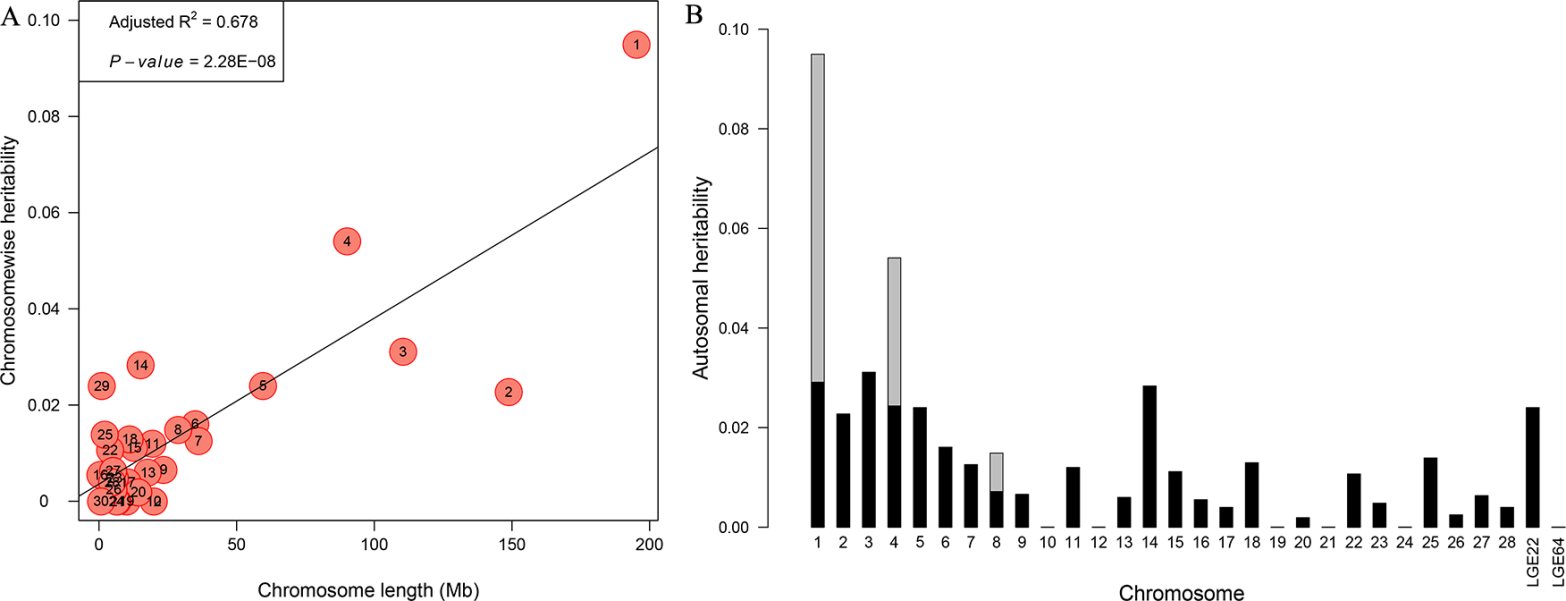
Genome partitioning of OW using a joint analysis. **A.** The estimated proportion of variance captured by each chromosome relative to its size. Solid lines are shown for linear regressions. The characters in circles are the chromosome numbers. **B.** Contributions of the GWAS SNPs partitioned by chromosome. The whole bars indicate the estimates of variance explained by each chromosome, of which the three gray bars represent the same values for the three leading loci.

We conducted a partitioning of genetic variation for OW because only OW showed the significant effects of individual chromosomes. We observed a strong, linear relationship between the estimate of the variance explained and chromosome length for OW (*R*^2^ = 0.68, **Fig 5A**). Additionally, GGA1, GGA4, and GGA8 explained 9.49% 5.40%, and 1.49%, respectively, of the phenotypic variance. After the three leading SNPs were fitted as covariates, the proportion of variance explained by GGA1, GGA4, and GGA8 decreased to 2.91%, 2.97%, and 0.78%, respectively (**Fig 5B**).

## Discussion

To our knowledge, this study is the first to detect potential genes that affect oviduct traits. Regarding the phenotypic data, the coefficient of variation of OL (13.91%) was lower than that of OW (23.37%). This may be because the length of the oviduct is relatively stable during the reproductive season, while the weight in relation to the laying hen is not. Additionally, the oviduct capacity remains unchanged during the laying season, while it increases when laying [29].

The heritabilities of OL and OW were moderate (0.354 and 0.392, respectively), and the genotypic effect of *rs318027552* for egg quality showed a consistent trend with oviduct traits, which means that the selection of these oviduct traits will greatly improve egg quality. Moreover, we found that the heritability of OW displayed a positive, linear correlation between the variance explained by each chromosome and the relative chromosome length, which is consistent with previous findings [30] and other traits in our population [13, 16]. In the genome partitioning analysis, GGA1 explained 2.91% of the phenotypic variance after fitting the three leading SNPs as covariates, and the SNP *rs318027552* that is located in this region explained the greatest amount of the phenotypic variation, which suggests that a significant SNP on GGA1 or a quantitate trait locus plays a key role in determining OW.

In our study, the characteristics of OL and OW were analyzed for the first time using a GWAS. Previously, no association had been found between SNPs and oviduct traits in chickens. We found 81 SNPs on GGA1 that were associated with OL, while 41, 8, and 3 SNPs on GGA1, GGA4, and GGA8, respectively, were associated with OW.

The significant SNPs on GGA1 are near the 170 Mb region that is also associated with egg weight and eggshell quality [16, 31], as well as body weight [32–34], and our findings revealed that this region also plays a crucial role in oviduct growth and albumen quality. The most significant SNP, *rs318027552*, is located 88.12 kb from *CKAP2*. *CKAP2* encodes cytoskeleton associated protein 2 that is involved in cell proliferation and cell survival [35], and which is essential for maintaining genomic stability [36]. A previous study showed that the oviduct mass remains unchanged during the laying period in zebra finches [37]; thus, *CKAP2* may participate in the hyperplasia and hypertrophy of the oviduct before the laying of the first egg.

The locus associated with OW is located from 74.03 to 76.70 Mb on GGA4. Yi and Sun [16, 31] reported that this region is also related to eggshell traits and egg weight including yolk, albumen, and eggshell weight, the latter two of which are generated mainly in the oviduct magnum and uterus, respectively. A non-synonymous mutation (*rs80668034*) is located downstream of *CCKAR*, which encodes cholecystokinin type-a receptor that is associated with appetite control [38]. Previous studies showed that *CCKAR* in chickens is responsible for up to a 19% difference in body weight at 12 weeks of age [39], and that it is related to growth traits [40, 41]. Moreover, studies by Xu [42] showed that *CCKAR* is associated with residual feed intake. During the laying period, egg formation is related to food consumption [43], and the feed intake increases after the first egg is laid. In the current study, the GG genotype of *rs80668034* had a higher albumen weight and eggshell weight; therefore, we hypothesize that *CCKAR* plays a role in the use of energy in the oviduct, as well as in egg formation.

Another SNP, *rs312570847*, is located near the gene *NCAPG* (encoding non-SMC condensin I complex, subunit G), which has pleiotropic effects on multiple traits, such as body weight [44] and residual feed intake in bovines [45] and withers height in horses [46]. In our population, we showed that *NCAPG* affected egg formation or eggshell weight in chickens [16, 17], while in this study, we demonstrated that *NCAPG* may be related to OW, which accounts for a larger proportion of the body weight during the reproductive season. Moreover, SNPs close to *CCKAR* and *NCAPG* also showed suggestive associations with OL (for example, *rs80668034*, *P* = 4.92E-06, **S1 Table**). In summary, *CCKAR* and *NCAPG* may affect OW by promoting albumen synthesis and eggshell formation during the reproductive season.

The nearest gene to *rs80715313* on GGA8 is *GORAB* that encodes SCY1-like 1-binding protein 1, which localizes predominantly to the trans Golgi network [47], where it performs important functions in the secretory and endocytic pathways [48]. The abundance of glandular tissue in the oviduct is involved in protein secretion [49], and ovalbumin and ovotransferrin are transported from the Golgi complex to the plasmalemma by microtubules [50], which combined with the result from the genotypic effect of *rs80715313* on albumen height, suggests that *GORAB* may have effects on the secretion of egg white proteins during egg formation. Moreover, findings obtained from human studies showed that autosomal recessive mutations of *GORAB* cause gerodermia osteodysplastica, which is characterized by wrinkly skin and osteoporosis [51]. Thus, *GORAB* may participate in calcium deposition during eggshell formation in the uterus.

The most meaningful locus, *rs312614123*, on GGA2 having a suggestive association with OL explained 2.05% of the CPV, and it contained a SNP located in the gene *IGFBP3*, which encodes insulin like growth factor (IGF) binding protein 3. *IGFBP3* is one of the proteins that binds IGF, a potent mitotic agent that is involved in many biological functions such as protein synthesis, cell differentiation, and ovary development [52]. Cell differentiation in the oviduct during maturation is stimulated by somatotropins, including IGF [53], which cause newly differentiated cells to undergo mitosis, thereby creating a clonal expansion of a particular cell type. The dramatic changes of the chicken oviduct are a result of a more rapid increase in the cell number and cell mass, and the IGF system has critical functions in growth regulation [54]. Moreover, *IGFPB3* was shown to be associated with early sexual maturation in chickens [55], which suggests that it may play a vital role in oviduct growth during sexual maturation, because the oviduct grows rapidly during this time.

## Conclusions

Our study is the first to show a novel association between potential genes and oviduct traits. The results showed that OL and OW are moderately heritable. Using a GWA analysis, we found 82 and 42 genome-wide, significant SNPs for OL and OW. The significant regions located from 167.7–169.9 Mb on GGA1 were associated with both OL and OW. Using a statistical analysis, we detected the candidate genes *CKAP2*, *CCKAR*, *NCAPG*, *GORAB*, and *IGFBP3*, which may play a role in determining oviduct characteristics. These findings increase our understanding of the molecular controls involved in oviduct development.

## Supporting information

S1 Table Genome-wide significant single-nucleotide polymorphisms (SNPs) for oviduct length (OL) and oviduct weight (OW), as determined by a univariate analysis in GEMMA. (XLSX)

S2 Table Genome-wide suggestive SNPs for OL and OW, as determined by a univariate analysis in GEMMA. (XLSX)

S1 Fig Genotype effect plot of rs80668034 for oviduct weight and egg quality. (TIF)

S2 Fig Genotype effect plot of rs80715313 for oviduct weight and egg quality. (TIF)
